# Investigating the clinical role and prognostic value of genes related to insulin-like growth factor signaling pathway in thyroid cancer

**DOI:** 10.18632/aging.205524

**Published:** 2024-02-07

**Authors:** Junyan Liu, Xin Miao, Jing Yao, Zheng Wan, Xiaodong Yang, Wen Tian

**Affiliations:** 1 Department of General Surgery, The First Medical Center, Chinese People’s Liberation Army (PLA) General Hospital, Beijing 100853, China

**Keywords:** insulin-like growth factor, thyroid cancer, survival, RiskScore, prognosis

## Abstract

Background: Thyroid cancer (THCA) is the most common endocrine malignancy having a female predominance. The insulin-like growth factor (IGF) pathway contributed to the unregulated cell proliferation in multiple malignancies. We aimed to explore the IGF-related signature for THCA prognosis.

Method: The TCGA-THCA dataset was collected from the Cancer Genome Atlas (TCGA) for screening of key prognostic genes. The limma R package was applied for differentially expressed genes (DEGs) and the clusterProfiler R package was used for the Gene Ontology (GO) and KEGG analysis of DEGs. Then, the un/multivariate and least absolute shrinkage and selection operator (Lasso) Cox regression analysis was used for the establishment of RiskScore model. Receiver Operating Characteristic (ROC) analysis was used to verify the model’s predictive performance. CIBERSORT and MCP-counter algorithms were applied for immune infiltration analysis. Finally, we analyzed the mutation features and the correlation between the RiskScore and cancer hallmark pathway by using the GSEA.

Result: We obtained 5 key RiskScore model genes for patient’s risk stratification from the 721 DEGs. ROC analysis indicated that our model is an ideal classifier, the high-risk patients are associated with the poor prognosis, immune infiltration, high tumor mutation burden (TMB), stronger cancer stemness and stronger correlation with the typical cancer-activation pathways. A nomogram combined with multiple clinical features was developed and exhibited excellent performance upon long-term survival quantitative prediction.

Conclusions: We constructed an excellent prognostic model RiskScore based on IGF-related signature and concluded that the IGF signal pathway may become a reliable prognostic phenotype in THCA intervention.

## INTRODUCTION

Thyroid cancer (THCA) as the most frequently diagnosed malignancy had high incidence in females worldwide [1]. According to the annual cancer study report, there had been 62,450 estimated new cases and 1,950 estimated deaths in 2015 [2], however, the estimated new cases have dropped to 43,720, and the deaths are 2,120 in 2023 [3], indicating the incidence of THCA has greatly decreased and the mortality is stable [4] causing tremendous threat to people’s life and health. The THAC originated from the parafollicular C cells or follicular epithelial cells [5], and its types and pathogenesis are diverse, meanwhile great progress has been made in developing diagnosis and intervention method for THCA control based on a variety of molecular targets and combination therapy [6]. Papillary thyroid carcinoma (PTC) is a common subtype of THCA characterized by relatively favorable prognosis after treatment, accounting for 80% of diagnosed cases [7]. Anaplastic thyroid cancer (ATC) accounted for 2% of diagnosed cases and is characterized by poor prognosis and the highest mortality, lacking effective treatments for ATC currently [8]. The surgery and radioactive iodine are primary treatment methods for THCA, and combined with the minimally invasive intervention and other auxiliary means to improve the cancer treatment effect. Mounting evidences indicated that the THCA had good intervention effect and relative high cure rate compared with other cancers [9, 10]. However, the patients with the specific subtypes, such as ATC, exhibited poor intervention effect and the tumor cells developed aggressive and drug-resistant properties causing enormous obstacles to clinical cure [11]. Therefore, identifying reliable molecular markers and developing effective prognostic model are beneficial for the risk stratification and design of precise treatment in THCA [12].

The insulin-like growth factor (IGF) signaling played a crucial role in regulating growth and development, and the disorder of IGF-signaling pathway is closely related to the pathogenesis and progression of many cancers [13]. Liver is the primary source of IGF, which binds to their corresponding receptors (IGFR) with different affinities [14]. The ligand-receptor binding phosphorylated the downstream substrates, such as Src homology collagen (SHC) and insulin receptor substrate (IRS) [15]. After that, the phosphorylated SHC activated the mitogen-activated protein kinase (MAPK) pathways for cell cycle regulation [16] and the IRS activated the phosphatidylinositol 3-kinase (PI3K)/AKT for RNA processing, protein translocation, cell proliferation, autophagy and apoptosis in malignancy [17] and IGF-I induced the anti-inflammatory cytokines interleukin (IL)-10 for cancer progression [18]. However, IGFs are also involved in the activating immune activities of lymphoid, myeloid, and hematopoietic cells through the autocrine, paracrine and endocrine ways [19]. IGF-I can restore the cell ability both T and B cells that undergone lethal irradiation [20] and promote mature B cells and plasmocyte proliferation [21]. Meanwhile, the IGF-I also enhanced the tumor necrosis factor α (TNF-α) and interleukin (IL)-8 cytokine expression in anti-cancer response [22]. Dysregulation of the IGF signaling system could affect the progression and prognosis of multiple cancers, and also has the potential to be applied to stem cell therapy [23]. Abnormal down-regulation of IGFBP expression has been proved to be suggestive of breast cancer risk in pregnant women during pregnancy and postpartum, and may also be involved in the suppression of the associated immune microenvironment [24]. In some studies, IGF-IR has been shown to be a breast cancer-related marker and contributes to the progression of epithelial-mesenchymal transformation (EMT) in tumors to a certain extent [25]. Abnormal IGF signaling system may be involved in the progression of diseases such as obesity and ovarian cancer by affecting nutrient absorption and energy metabolism [26]. Both ligand and receptor systems of IGF were overexpressed in THCA tissue [27]. The report found that IGF-I is relatively actively expressed in some subtypes of THCA, such as PTC, which can stimulate the phosphorylation of corresponding receptors and induce tumor cells to accelerate the mitosis process [28]. At present, a number of researches have investigated the potential correlation between IGF-related genes and breast cancer prognosis, and the IGF system may also affect THCA prognosis at the genomic level [29]. Thus, we aimed to develop a useful risk prognosis model based on the IGF-related signatures.

This project will explore the clinical features of genes correlated with IGF signaling pathway in THCA, and assess the prognostic significance of IGF-correlated genes. We extracted and screened IGF-related genes according to TCGA and MSigDB to acquire key genes affecting prognosis, and designed and validated a RiskScore model to forecast the prognosis and survival of patients. Additionally, we also analyzed the clinicopathological features, mutation features, immune microenvironment and related biological pathway changes of different THCA patients with the model results.

## MATERIALS AND METHODS

### Data acquisition and preprocessing

(1) The RNA-Seq data of TCGA-THCA were downloaded from The Cancer Genome Atlas (TCGA) database through the GDC API tool [30, 31], in which 502 tumor samples and 58 normal samples were included in the RNA-Seq data, the patients missing the survival time, status, and clinical follow-up information were removed from this study, lastly, a total of 487 tumor samples were obtained.

(2) The IGF related gene set was downloaded from the Molecular Signatures Database (MsigDB, https://www.gsea-msigdb.org/gsea/msigdb), and 40 IGF-related genes were obtained [32], which are listed in Supplementary Materials (Supplementary Table 1).

### Identification and enrichment analysis of DEGs

We identified the differentially expressed genes (DEGs) between the tumor and adjacent normal samples by using limma R packages (setting |FC|>1.5 and FDR<0.05) [33]. Then the IGF score of tumor samples was calculated by using the GSVA R package [34], and the volcano map was made by spearman correlation analysis of Hmisc package to acquire the DEGs associated with IGF score (|R|>0.4, p<0.05) [35]. Next, we used the clusterProfiler package for GO and KEGG enrichment analysis of DEGs [36].

### Screening of key genes

(1) We randomly divided the TCGA-THCA dataset into the training set and the test set with 1:1 proportion, and compared the difference between the groups by using the Chi-square test [37].

(2) The survival R package was used to perform the univariate Cox proportional risk regression analysis for IGF score related DEGs in training set, the p < 0.05 as the filtering threshold [38].

(3) The univariate Cox regression analysis was applied for the significant prognostic gene. Then, the Least absolute shrinkage and selection operator (LASSO) compression was performed by using glmnet R package to reduce the number of candidate genes [39]. The model was designed using 10-fold cross-validation and the confidence intervals under each lambda were researched [40]. When the model is optimized, the gene is chosen as the target gene for the next step. The multivariate Cox regression analysis with the stepwise regression was used to determine the final model genes and calculate the regression coefficient [41].

### Constructing and verification of RiskScore model

We calculated the RiskScore for each sample using the following formula:


RiskScore=∑βi×Expi

(i representing gene expression level and the β is the Cox regression coefficient).

Based on the median RiskScore, the patients were classified into high- and low- RiskScore categories. For prognosis analysis, survival curves were plotted using the Kaplan-Meier technique, and the significance of differences was assessed using the logarithmic rank test [42]. The ROC analysis with the Area Under Curve (AUC) value was performed using the R software timeROC package [43].

### RiskScore analysis of comprehensive clinicopathological features

The expression of key prognostic model genes in the TCGA-THCA cohort and the RiskScore groups were compared, and a heatmap of clinicopathological characteristics was produced using the pheatmap tool. Riskscore and clinicopathological characteristics were subjected to univariate and multivariate Cox regression analysis in order to identify significant prognostic variables. In the TCGA-THCA cohort, the decision tree was built based on the patients’ age, sex, clinical grade of T, N, and M Stages and RiskScore grouping. Subsequently, several risk subgroups were found, and the variations in overall survival across subgroups were analyzed.

### Developing of nomogram

Using the rms R package, we integrated Riskscore and clinicopathological variables to create a nomogram that quantified the patient’s risk assessment and survival probability [44]. Further, we use Calibration curve and Decision curve to assess the prediction accuracy of the model [45].

### Gene set enrichment analysis (GSEA)

In order to observe the relationship between RiskScore and biological function of different samples, we chose the TCGA dataset for analysis. Using H.LL.v7.5.1.symbols.gmt as gene set, R software GSVA package was used for ssGSEA [46], and the scores of each sample in different functions were computed. Then the correlation between RiskScore and channel score was calculated by spearman method.

### Analysis of tumor microenvironment

We performed CIBERSORT and MCP-counter algorithms to estimate immune infiltration difference between varying risk groups [47]. Based on the expression of marker genes of immune cells, the CIBERSORT algorithm assessed the degree of immune infiltration through calculating the immune infiltration score of 22 immune cells in varying risk groups. Meanwhile, we calculated the correlation between the RiskScore and the immune infiltration by using the spearman method. The MCP-Count algorithm used for the immune infiltration score of other 10 immune cells, and its correlation with the RiskScore were also calculated. In addition, we analyzed the correlation between the common immune checkpoints and the RiskScore. The higher tumor stemness represented the stronger potential of tumor cell renewal and differentiation and the poor progression-free survival (PFS) of patients, we further calculated the correlation between the RiskScore and the tumor stemness index.

### Analysis of mutation characteristics

First, the tumor mutation burden (TMB) of the RiskScore groups was compared. Next, the R software Survminer package was performed to divide the TCGA-THCA tumor samples into two groups with high and low TMB according to the optimal TMB threshold, compare the survival difference between the two groups, and analyze the synergistic effect of TMB and RiskScore.

The THCA RNA-seq data of normal (58) and tumor-samples (502) were processed by mutect2 software to obtain the THCA mutation dataset, which were further used for screening the genes with significant high frequency mutations by using the fisher rest (p< 0.05 and mutation frequency > 3) [48]. Then we performed fisher test to screen genes with high mutation frequency in each group (p < 0.05), and finally acquired key mutant genes. Somatic alterations in the tumor-associated pathways in the group were then assessed, including Hippo, PI3K, WNT, NOTCH, MYC, NRF2, TGF-β, TP53, RTK-RAS, and Cell-Cycle.

### Statistical analysis

This study mainly performs R language for statistical analysis. The wilcoxon rank sum test was used for the significance of difference between two sets of continuous variables, the spearman method was used for correlation analysis (p-value < 0.05 as a statistically significant, * p-value <0.05, ** p-value <0.01, *** p-value <0.001 and “ns” is no significant difference).

### Data availability statement

The datasets generated during and analyzed during the current study are available from the corresponding author on reasonable request.

## RESULTS

### Screening and enrichment of DEGs

**Figure 1:**
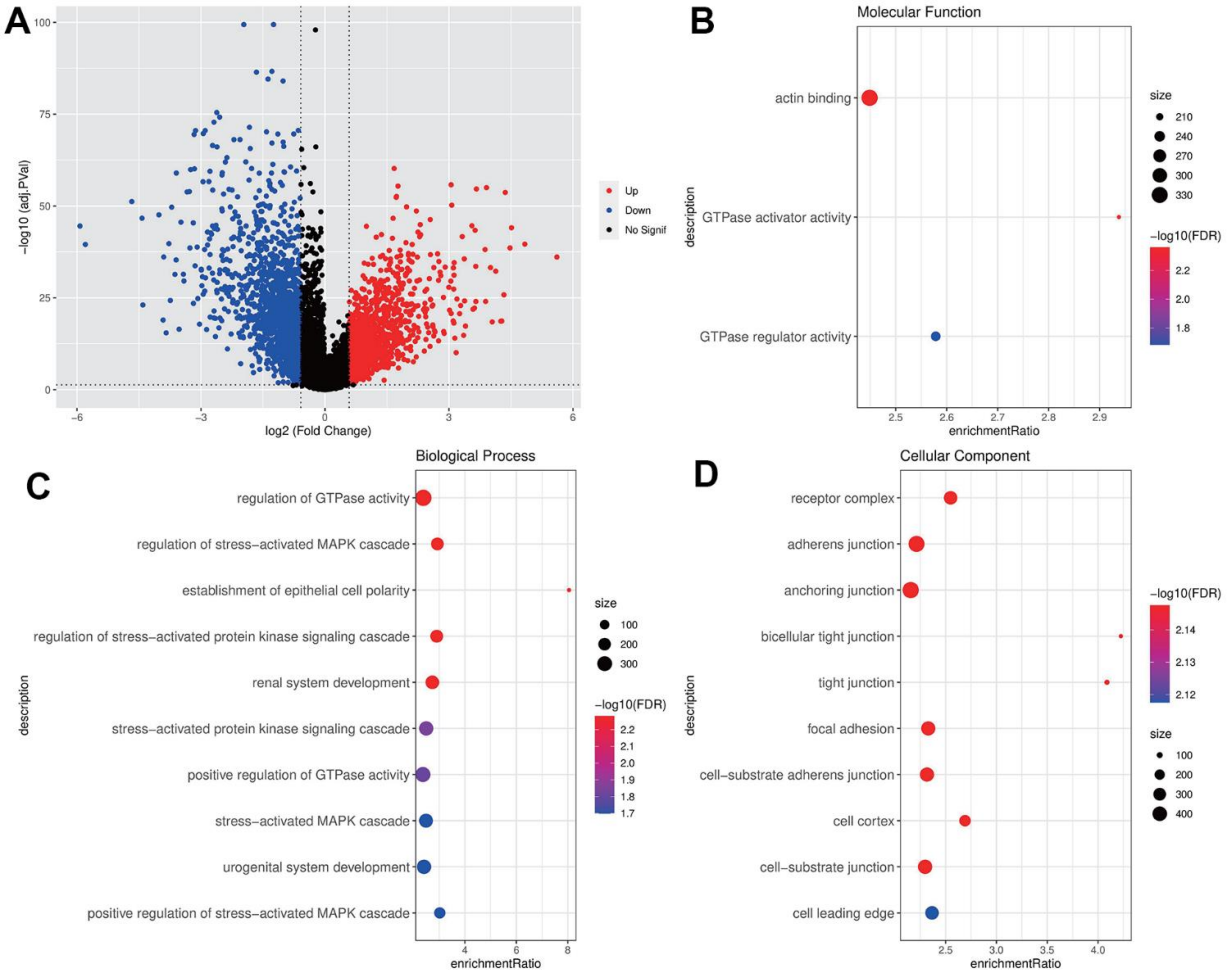
**Screening and enrichment of DEGs.** (**A**) DEGs volcano maps of THCA and normal samples in TCGA dataset; (**B**–**D**) GO enrichment analysis bubble chart of IGF score related DEGs (MF, BP, CC).

A total of 3211 DEGs were acquired by difference analysis between THCA samples and normal samples of TCGA, in which 721 DEGs were closely correlated to the IGF score (Figure 1A). The Molecular Function of GO enrichment analysis indicated that these genes were closely associated with the actin binding pathway (Figure 1B), the biological process of GO indicated that these genes were closely associated with the activation of regulation of GTPase activity and stress-activated MAPK cascade pathways (Figure 1C), the cellular component of GO displayed that the most of receptor complex, adherens junction, focal adhesion and cell-substrate adherens junction pathway were activated (Figure 1D), implying these genes could be may be closely related to epithelial cell transformation or cancer metastasis.

### Establishment of RiskScore model

**Figure 2:**
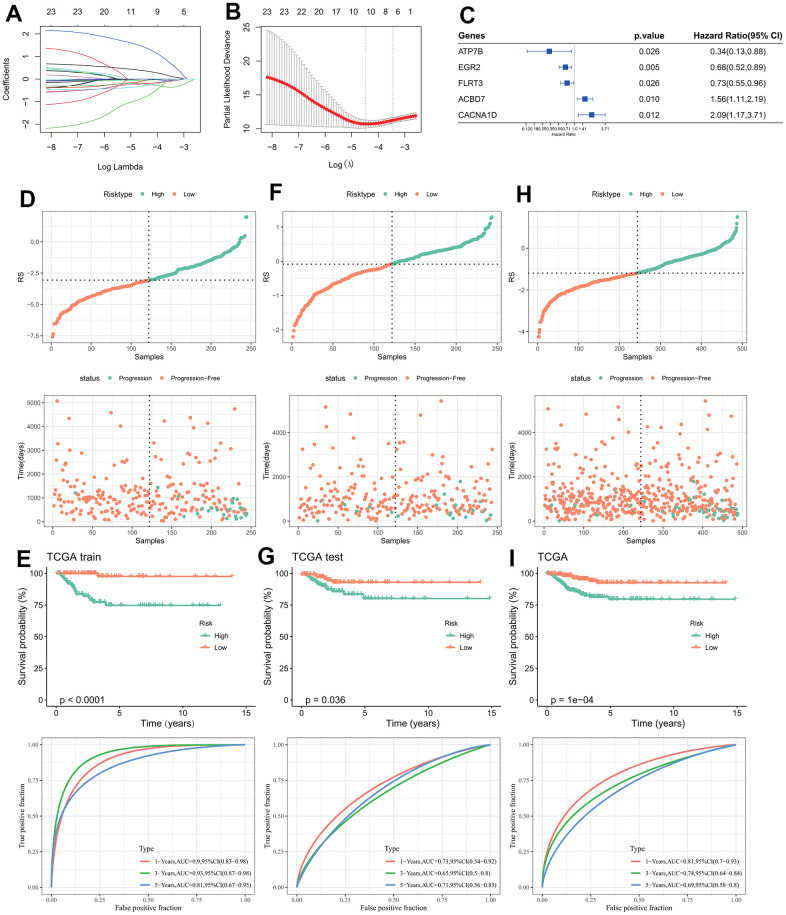
**Design and validation of RiskScore model.** (**A**) The trajectory of each independent variable changing with lambda; (**B**) Confidence interval under lambda; (**C**) Forest map of characteristic gene prognosis; (**D**, **E**) RiskScore of TCGA training set, ROC curve and KM curve of RiskScore model; (**F**, **G**) RiskScore of TCGA test set, ROC curve and KM curve of RiskScore model; (**H**, **I**) RiskScore of TCGA data set, ROC curve and KM curve of RiskScore of model.

Chi-square test results between the training set and test set indicated no significant difference in clinicopathologic features groups, indicating that our grouping was random and reasonable (p < 0.05, Table 1). 25 genes were retained after IGF score related EDGs filtering. Lasso regression analysis indicated that the number of independent variable coefficients approaching 0 rose together with the progressive increase in lambda (Figure 2A). Ten genes at lambda=0.01147 were chosen as the target genes for the following phase after analyzing the confidence interval under each lambda (Figure 2B). After stepwise regression, five genes were identified as key genes affecting prognosis, namely EGR2, ATP7B, CACNA1D, ACBD7, and FLRT3 (Figure 2C).

**Table 1:** Clinical information of TCGA dataset.

**Characteristics**	**TCGA-train (N=244)**	**TCGA-Test (N=243)**	**Total (N=487)**	**p-value**	**FDR**
**Age**				0.08	0.44
<=46	135(27.72%)	114(23.41%)	249(51.13%)		
>46	109(22.38%)	129(26.49%)	238(48.87%)		
**Gender**				0.78	1
FEMALE	177(36.34%)	180(36.96%)	357(73.31%)		
MALE	67(13.76%)	63(12.94%)	130(26.69%)		
**T.stage**				0.01	0.08
T1	73(14.99%)	68(13.96%)	141(28.95%)		
T2	74(15.20%)	89(18.28%)	163(33.47%)		
T3	92(18.89%)	68(13.96%)	160(32.85%)		
T4	5(1.03%)	16(3.29%)	21(4.31%)		
Unknow	0(0.0e+0%)	2(0.41%)	2(0.41%)		
**N.stage**				0.07	0.44
N0	108(22.18%)	116(23.82%)	224(46.00%)		
N1	117(24.02%)	96(19.71%)	213(43.74%)		
Unknow	19(3.90%)	31(6.37%)	50(10.27%)		
**M.stage**				0.32	1
M0	146(29.98%)	129(26.49%)	275(56.47%)		
M1	3(0.62%)	3(0.62%)	6(1.23%)		
Unknow	95(19.51%)	111(22.79%)	206(42.30%)		
**Stage**				0.33	1
I	146(29.98%)	134(27.52%)	280(57.49%)		
II	21(4.31%)	30(6.16%)	51(10.47%)		
III	54(11.09%)	50(10.27%)	104(21.36%)		
IV	23(4.72%)	27(5.54%)	50(10.27%)		
Unknow	0(0.0e+0%)	2(0.41%)	2(0.41%)		
**PFS**				1	1
Progression-free	221(45.38%)	221(45.38%)	442(90.76%)		
Progression	23(4.72%)	22(4.52%)	45(9.24%)		

The final model formula is as follows:

RiskScore=(-0.228*EGR2)+(-1.491*ATP7B)+0.888*CACNA1D+0.336*ACBD7+(-0.082*FLRT3)

The three data sets (TCGA training set, TCGA test set, and TCGA data set) were divided into high and low RiskScore group according to the median RiskScore (Figure 2D, 2F, 2H). The KM curves of the three data sets all indicated that the patient’s survival probability had significantly difference, in which patients in the high-risk groups were associated with poor prognosis. ROC curves of the three datasets indicated that AUC values were 0.9, 0.73 and 0.81 in the first year, 0.93, 065 and 0.74 in the third year, and 0.81, 0.71 and 0.69 in the fifth year (Figure 2E, 2G, 2I), the higher AUC value indicated the RiskScore had excellent classification performance in long- and short-prognostic prediction [49].

### Analysis of RiskScore combined with clinicopathological features and construction of the nomogram

**Figure 3:**
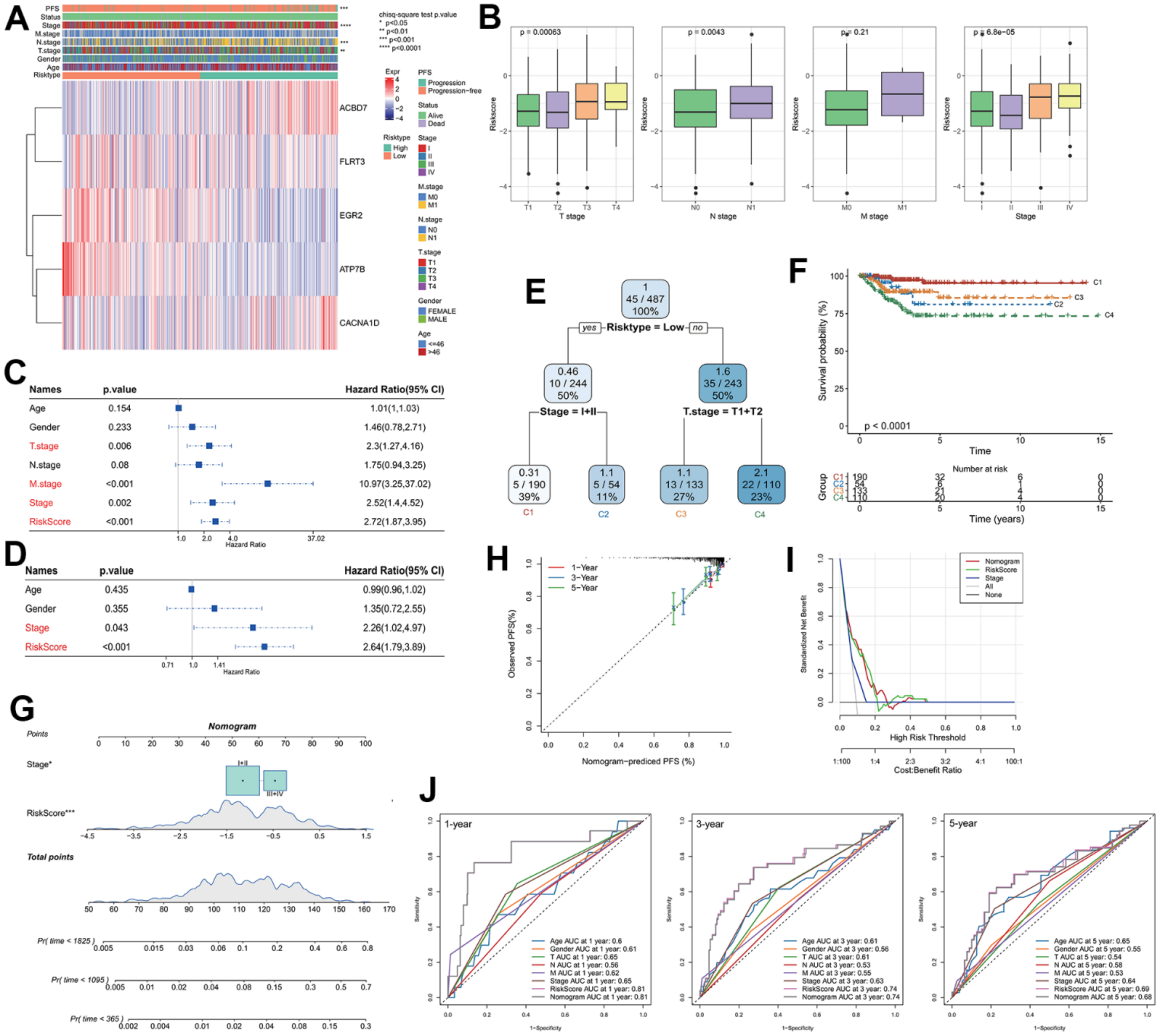
**Analysis of RiskScore combined with clinicopathological and construction of the nomogram.** (**A**) Relationship between model gene expression and clinicopathological features; (**B**) Differences in RiskScore between clinicopathological grades; (**C**) Results of single-factor Cox analysis of clinicopathological features; (**D**) Multivariate Cox analysis results of clinicopathological features; (**E**) Using RiskScore and clinicopathological features to construct the survival decision tree; (**F**) Differences in survival rates among the four subgroups; (**G**) RiskScore combined with clinicopathological features to establish a nomogram; (**H**) Calibration curves for 1, 3 and 5 years of the nomogram; (**I**) Decision curve of the nomogram; (**J**) The ROC curves of the nomogram and a variety of clinicopathological features at 1, 3 and 5 years.

The expression of key genes was compared with the clinicopathological characteristics of RiskScore groups. It was indicated that there were significant differences in T.tage, N.tage, Stage and PFS between RiskScore groups. ACBD7 and CACNA1D as risk factors were significantly expressed in the group with high RiskScore, while FLRT3, EGR2 and ATP7B as protective factor were more actively expressed in the group with low RiskScore (Figure 3A). The RiskScore increased with the increasing clinicopathological grade (Figure 3B). Univariate and multivariate Cox regression analysis indicated that Riskscore and Stage were significant prognostic independent factors (Figure 3C, 3D). We incorporated RiskScore and several clinical features into a decision tree model, which is mainly used to construct a risk hierarchical classifier. Through the decision tree model, the patients were divided into four different clusters (C1, C2, C3, C4), we found that RiskType’s classification ability was superior to other clinical information, followed by T-stage and Stage feature (Figure 3E), indicating the RiskScore is an important risk decision factor. There were significant differences in overall survival among the risk subgroups, with the C1 subgroup having the highest survival rate, followed by C3, C2, and C4 (Figure 3F), indicating that the RiskScore could be a reliable classification indicator.

A nomogram was created using RiskScore and additional clinicopathological characteristics, and the RiskScore showed the greatest influence on the prediction of survival rate (Figure 3G). The calibration curves for the calibration points at 1-, 3-, and 5- years are seen to be quite near to the standard calibration curve, demonstrating the nomogram’s strong predictive ability (Figure 3H). The results of decision curves and ROC curves of each clinicopathological features indicated that both nomogram and RiskScore showed the strongest predictive power compared with other clinicopathological features (Figure 3I, 3J), these results indicated that the nomogram can quantify the survival probability of the patient effectively and benefit the clinical patients.

### Differences in immune microenvironment between RiskScore groups

**Figure 4:**
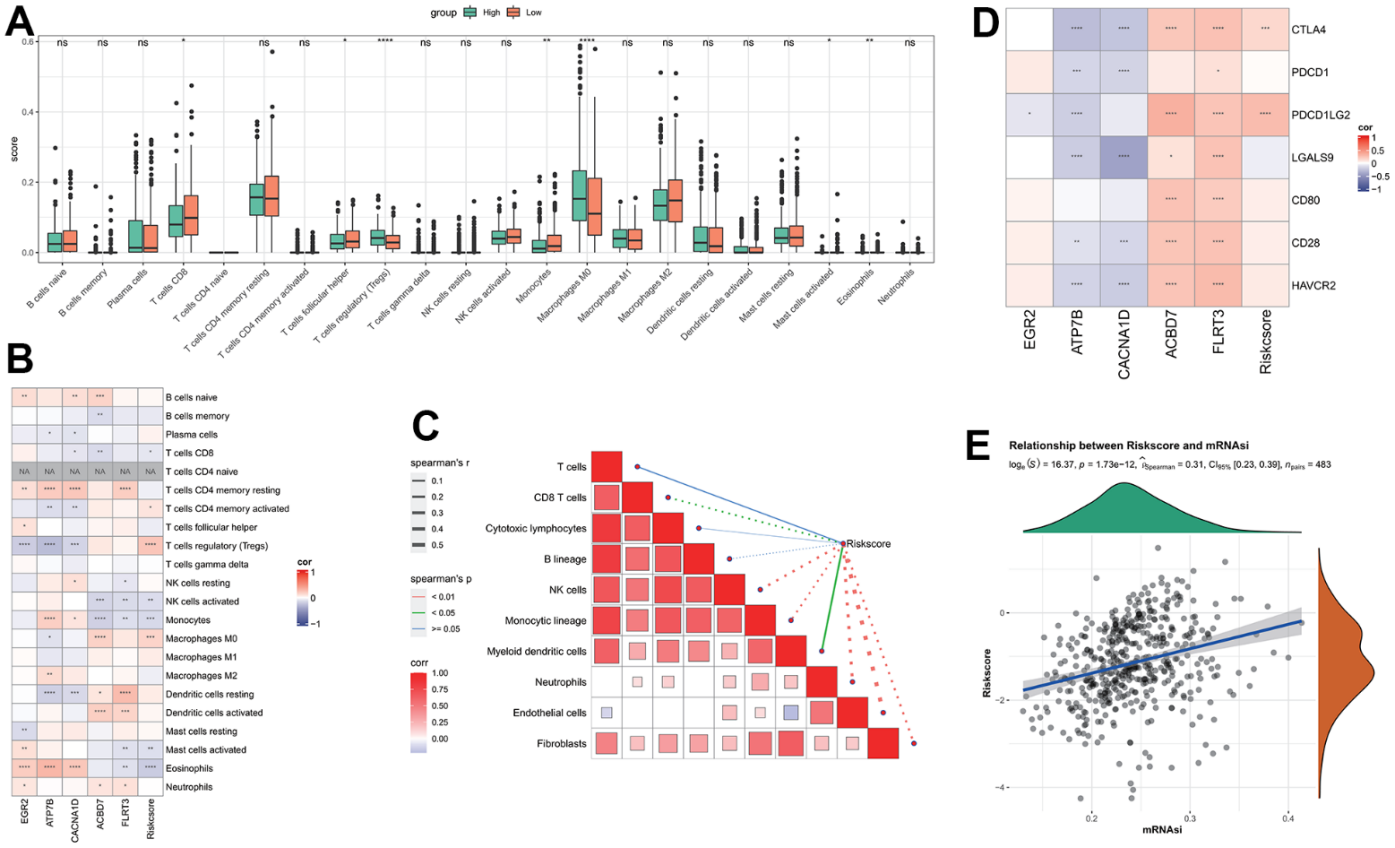
**Analysis of immune features between RiskScore groups.** (**A**) The difference of immune infiltration by CIBERSORT analysis between RiskScore groups; (**B**) The correlation between RiskScore and CIBERSORT’s immune score; (**C**) The correlation between RiskScore and immune score analyzed by MCP-Count; (**D**) RiskScore is associated with immune checkpoint genes; (**E**) Correlation between RiskScore and tumor stemness index.

CIBERSORT analysis indicated that there were significant differences between the seven immune cells in different groups. The proportions of CD8 T cells, T cells follicular helper, Monocytes and Activated mast cells in the low RiskScore group were significantly higher than those in the high RiskScore group (Figure 4A), indicating that the low-risk patients had more strong immune ability against tumor cells. The correlation between RiskScore and CIBERSORT immune infiltration score showed that the RiskScore was significantly negative correlation to most immune cell infiltration score (Figure 4B), these results are consistent with the results of 22 immune cells infiltration in high-risk patients, the patients with higher RiskScore are associated with worst immune cell infiltration. A substantial inverse relationship between RiskScores and the scores of the majority of immune cells was found by the MCP-Count algorithm, while several model genes exhibited obviously positive correlation to the immune cell infiltration score, such as the ABCD7 and ELRT3 are closely associated with the activated NK cells (Figure 4C). RiskScore, FLRT3, and ACBD7 were found to be positively correlated to most immune checkpoint genes, whereas ATP7B and CACNA1D were shown to be negatively correlated with most of immune checkpoint genes (Figure 4D), suggesting that FLRT3 and ACBD7 are closely associated with the immunotherapy effect and can be developed as a combination target for immunotherapy [35].

The correlation results of RiskScore and THCA tumor stemness index indicated that there was a significant positive correlation between RiskScore and mRNAsi (Figure 4E).

### Differences in mutation features between groups

**Figure 5:**
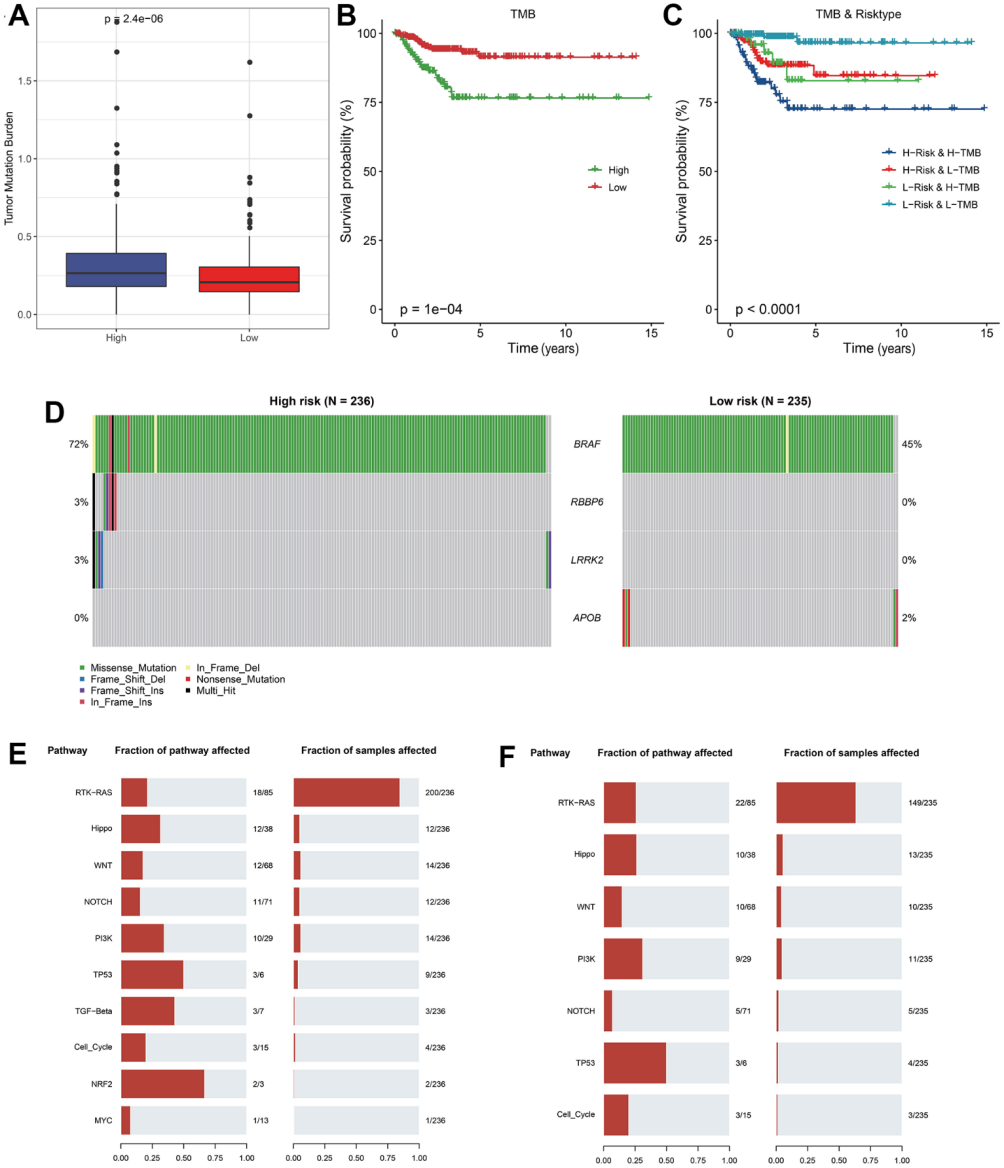
**Analysis of mutation features between RiskScore groups.** (**A**) TMB differences between RiskScore groups; (**B**) KM curve between high and low TMB groups in TCGA cohort; (**C**) KM curve of high and low TMB groups and high and low RiskScore of TCGA cohort; (**D**) Significantly different mutated genes between RiskScore groups; (**E**) Mutation frequency of tumor pathway genes and proportion of affected samples in the high RiskScore group; (**F**) Frequency of mutation of tumor pathway genes and proportion of affected samples in low RiskScore group.

The TMB comparison in varying risk groups indicated that the TMB of the high RiskScore group was higher (Figure 5A), the survival probability was significantly lower in the high TMB group (Figure 5B), implying the high-risk patients had poor prognosis after treatment. The synergistic effect of TMB and RiskScore showed that regardless of the high TMB subgroup or the low TMB subgroup, the prognosis of patients in the low RiskScore group was always better than that in the high RiskScore group, indicating that the TMB status did not hinder the predictive effect of RiskScore (Figure 5C).

Four genes with high frequency mutation in each subtype were screened. The mutation features of genes in different groups indicated that BRAF gene had a significant mutation frequency in the high RiskScore group, and most of them were missense mutations. Somatic cell changes in tumor-related pathways in the high and low RiskScore groups were evaluated. It was found that the mutation rate in the high RiskScore group was the highest, and the proportion of affected samples was the largest (Figure 5E, 5F), implying that the highly mutation of these classic cancer-related pathway conferred tumor cell with more strong survival and immune suppressive ability in tumor microenvironment.

### Correlation between RiskScore and biological pathways

**Figure 6:**
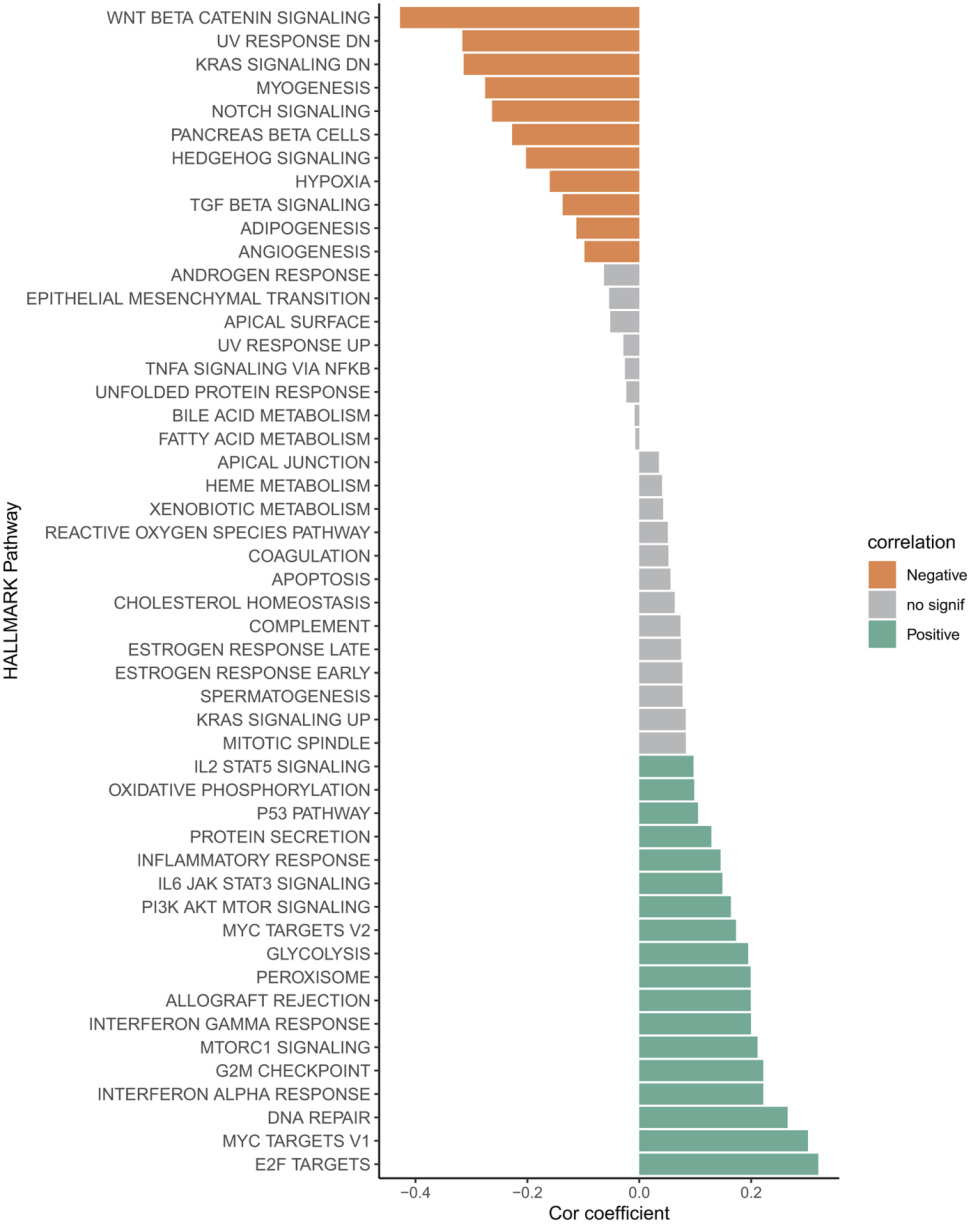
Correlation between RiskScore and HALLMARK channel score.

The correlation between RiskScore and pathway score was estimated, and it was found that Wnt-β catenin signaling, UV response, KRAS signaling and Notch signaling, Hypoxia, TGF-β signaling and angiogenesis pathways were significantly negative correlated to the RiskScore (Figure 6). However, the E2F targets, Myc targets-v1/v2, interferon alpha and gamma response, G2M checkpoint, mTORC1 signaling, peroxisome, glycolysis and PI3K AKT MTOR signaling pathways were significantly positive correlated to the RiskScore (Figure 6), implying the tremendous heterogeneity existed in varying risk patients mediated by the different cancer activation pathway.

## DISCUSSION

Thyroid cancer (THCA) is the most common endocrine malignancy characterized by highly incidence and invasion in female worldly. Many researches have shown that IGF signaling pathway is closely related to the THCA, and IGF system can interact with a variety of biological pathways to affect the growth and spread of tumor tissue [50]. In addition, the serum of THCA patients was detected, and the IGF-1R concentration was generally elevated in the serum of the subjects [51]. In the research of Piotr Tomasz Wysocki, it was found that the reduced expression level of DIRC3, a long non-coding RNA gene, can enhance the susceptibility of THCA cells to IGF1 stimulation, promote Akt signaling by down-regulating IGFBP5 protein, and increase the invasion ability and spread of THCA cells [52]. Clinical studies have indicated that IGF-blocking therapy, including monoclonal antibodies and IGF-related inhibitors, may be a new strategy for cancer treatment [53]. Lv et al. claimed that IGF can promote THCA stemness and increase tumor cell invasiveness by activating PI3K/AKT/mTOR signaling pathway, and blocking IGF signaling pathway can inhibit this process [54]. IGFBP-3 is a regulatory protein of p53 tumor suppressor factor, which can destroy some important life processes including transcription of various cancer cells and play a role in tumor inhibition [55]. Therefore, it is of great significance for us to focus on and identify key IGF-related genes for clinical research and prognostic value mining of THCA.

In this study, we acquired five key genes affecting prognosis, in which the CACNA1D and ACBD7 were regarded as risk factors, while the EGR2, FLRT3 and ATP7B were regarded as protective factors in model. Early growth response proteins (EGRs) are a family of multifunctional transcriptional regulatory proteins, of which EGR2 is one of the most studied. Levels of EGR2 are relatively low in THCA tissues, but overexpression of EGR2 can prevent the growth and spread of cancer cells [56]. EGR2 can be targeted by MiR-224-5p to promote the proliferation and invasion of THCA [57]. Some researches have found that EGR2 is the target gene of miR-25 that can promote the proliferation of cancer cells, and knocking down EGR2 will promote the proliferation and spread of tumor tissues [58]. ATP7B is a type of copper effector transporter, which is a key protein in maintaining copper metabolism and copper homeostasis in cells, and is also associated with some cancer prognostic effects [59]. Cisplatin and carboplatin are common chemotherapy drugs, and ATP7B can mediate the resistance of cancer cells to platinum-based chemotherapy drugs, helping to alleviate the stress of cancer cells, resulting in poor benefits of traditional chemotherapy for patients [60]. Mengdi Yang et al. found in the study of colorectal cancer that the up-regulated expression of FLRT3 can inhibit the growth and invasion of tumor cells, leading to the apoptosis of tumor cells, but the down-regulated expression of FLRT will lead to the opposite result, and patients will have a poor prognosis [61]. CACNA1D is a key protein encoding calcium ion channel subunits, which can effectively participate in regulating calcium ion concentration and maintaining intracellular homeostasis [62]. In gastric cancer studies, it was found that CACNA1D was regulated by tRNA derivatives to participate in MAPK signaling pathway transmission, inhibiting the growth and metastasis of tumor cells [63]. ACBD7 is a member of the multigene family containing Acyl-CoA binding domain (ACBD), which plays an important role in life activities such as energy metabolism and nutrient uptake [64]. ACBD7 can be regulated by transcription factors to promote muscle development and lipid metabolism, but cancer-related studies are lacking [65]. ACBD3, from the same family as ACBD7, has been found to be up-regulated in a variety of tumor tissues and may be responsible for poor breast cancer prognosis [66, 67]. The function of these genes promoting or inhibiting cancer progression in multiple cancers had been reported, indicating that these model genes had higher reliable prognostic value supporting model construction.

When we analyzed the correlation between RiskScore and THCA tumor stemness index, we discovered that there was a significant positive correlation between Riskcsore and mRNAsi. Studies have indicated that high levels of mRNAsi show that cancer cells are more active in various life activities, have stronger potential for differentiation and invasion, and increase tumor drug resistance [68, 69]. This may be the reason for the poor immune cell scores and immune infiltration in most patients with high RiskScore.

The mutation features analysis the BRAF had the highest mutation frequency in low- and high-risk patients. Integrative clinical genomics had revealed hundreds of key genes that occurred significant mutation in tumorigenesis, such as the cyclin-dependent kinase inhibitor 2A (CDKN2A), tumor protein p53 (TP53), and retinoblastoma (RB1) phosphatidylinositol-4,5-bisphosphate 3-kinase catalytic subunit alpha (PIK3CA) [70, 71]. The BRAF is proto-oncogene [72] and has been found to be mutated in a variety of cancers, including non-small cell lung cancer, colorectal cancer, and melanoma to mediate the oncogenetic phenotype [73–75]. Therefore, the BRAF could be a useful marker of THCA occurrence. The CD8 T cell and Monocytes are significantly infiltrated in the low-risk groups and contributed to eliminate the tumor cells [76], however, the macrophages M0 are significantly enriched in the low-risk group. It is well-known that the macrophages contribute significantly to pathogen eliminating and cancer killing at early-stage of the disease [77], while the tumor cells released several chemokines (CCL2, CCL5 and CXCL12) and cytokines (VEGF and Csf1) to recruit more monocytes that are primitive macrophage M0 and they promote differentiation to macrophages M2 at advanced tumor-stages [78]. The abundant anti-inflammatory macrophages M2 infiltration is not conducive to the anti-tumor function of other immune cells, thus M1 macrophage inducers may be more beneficial for cancer treatment in high-risk patients [79], meanwhile based on the significant positive correlation, the overexpression of ABCD7 and FLRT3 could enhance the NK cell activation for tumor cell killing.

In addition, the cancer progression in varying risk patients appears to be mediated by different cancer activation pathways. The Wnt/β catenin is a growth stimulating factor promoting cell proliferation and affecting cell cycle regulation [80], the downregulation Wnt/β-catenin signaling often leads to multiple diseases [81]. Oncogenic KRAS mediated KRAS signaling pathway is crucial for cell growth, differentiation and survival, its continuous activation is closely associated with the development of breast cancer, colon cancer and pancreatic cancer [82–84]. The hypoxia is an inducible factor of angiogenesis supporting tumor growth and proliferation [85]. These typically cancer signaling pathways were significantly negatively correlated to the RiskScore, suggesting these pathways were significantly activated in the low-risk patients, thus we can develop the corresponding intervening scheme targeting these signaling pathways for low-risk patients’ precise treatment. Similarly, the cell cycle related pathways E2F targets, Myc targets-v1/v2 and G2M checkpoint [86, 87], the metabolism-related pathways mTORC1 signaling, peroxisome and glycolysis and PI3K AKT mTOR signaling [88, 89], could be intervention targeting for high-risk patients’ precise treatment.

Overall, we screened five IGF-related prognostic genes and constructed a reliable RiskScore model for patient’s risk stratification, this study is expected to help clinicians make more effective treatment decisions and design advisable personalized therapeutic schedule for patients. However, our research also has major limitations. All the data came from the database with a single sample size and lack of complete clinicopathological information. Moreover, both the training set and the validation set were from the same dataset. In the future, we hope to expand the scope of cancer research and the number of valid samples, and integrate more complete clinicopathological information to carry out model design. More research is needed to uncover the specific pathogenesis and optimal treatment of THCA.

## CONCLUSIONS

We screened IGF related genes and acquired five key genes that affect prognosis: EGR2, ATP7B, CACNA1D, ACBD7 and FLRT3. A RiskScore model was designed based on 5 key genes. Combined with nomogram verification, this model could accurately predict the prognosis risk of THCA patients. Our results may contribute to the clinical treatment of THCA patients and further study of tumor mechanisms.

## Supplementary Material

Supplementary Table 1
