# Supplementary material for: Investigating the clinical role and prognostic value of genes related to insulin-like growth factor signaling pathway in thyroid cancer
Source: Aging (Albany NY). 2024 Feb 7;16(3):2934–52. doi: 10.18632/aging.205524 (PMC10911384; doi:10.18632/aging.205524)
Supplement: Supplementary Table 1 [file aging-16-205524-s001.pdf]

SUPPLEMENTARY TABLE

Supplementary Table 1. The list of 40 IGF-related genes is showed in.

CSNK2A1
ELK1
FOS
GRB2
HRAS
IGF1
IGF1R
IRS1
JUN
MAP2K1
MAPK3
MAPK8
PIK3CA
PIK3CG
PIK3R1
PTPN11
RAF1
RASA1
SHC1
SOS1
SRF
AKT1
BAD
BCAR1
CRK
CRKL
GRB10
IRS2
NCK2
PDPK1
PRKCD
PRKCZ
PRKD1
PTK2
PTPN1
PXN
RACK1
RPS6KB1
YWHAE
YWHAZ
